# Time trend and age-period-cohort effect on kidney cancer mortality in Europe, 1981–2000

**DOI:** 10.1186/1471-2458-6-119

**Published:** 2006-05-03

**Authors:** Napoleón Pérez-Farinós, Gonzalo López-Abente, Roberto Pastor-Barriuso

**Affiliations:** 1 Department of Epidemiology, Madrid Public Health Institute, Julián Camarillo 4B, 28037 Madrid, Spain; 2 National Center for Epidemiology, Carlos III Institute of Health, Sinesio Delgado 6, 28029 Madrid, Spain

## Abstract

**Background:**

The incorporation of diagnostic and therapeutic improvements, as well as the different smoking patterns, may have had an influence on the observed variability in renal cancer mortality across Europe. This study examined time trends in kidney cancer mortality in fourteen European countries during the last two decades of the 20th century.

**Methods:**

Kidney cancer deaths and population estimates for each country during the period 1981–2000 were drawn from the World Health Organization Mortality Database. Age- and period-adjusted mortality rates, as well as annual percentage changes in age-adjusted mortality rates, were calculated for each country and geographical region. Log-linear Poisson models were also fitted to study the effect of age, death period, and birth cohort on kidney cancer mortality rates within each country.

**Results:**

For men, the overall standardized kidney cancer mortality rates in the eastern, western, and northern European countries were 20, 25, and 53% higher than those for the southern European countries, respectively. However, age-adjusted mortality rates showed a significant annual decrease of -0.7% in the north of Europe, a moderate rise of 0.7% in the west, and substantial increases of 1.4% in the south and 2.0% in the east. This trend was similar among women, but with lower mortality rates. Age-period-cohort models showed three different birth-cohort patterns for both men and women: a decrease in mortality trend for those generations born after 1920 in the Nordic countries, a similar but lagged decline for cohorts born after 1930 in western and southern European countries, and a continuous increase throughout all birth cohorts in eastern Europe. Similar but more heterogeneous regional patterns were observed for period effects.

**Conclusion:**

Kidney cancer mortality trends in Europe showed a clear north-south pattern, with high rates on a downward trend in the north, intermediate rates on a more marked rising trend in the east than in the west, and low rates on an upward trend in the south. The downward pattern observed for cohorts born after 1920–1930 in northern, western, and southern regions suggests more favourable trends in coming years, in contrast to the eastern countries where birth-cohort pattern remains upward.

## Background

Kidney cancer incidence and mortality rates have increased during the last years in different countries [1-4]. Renal cancer caused 29,546 deaths in men and 16,682 in women in the European Union in 1998[5], and accounts for approximately 3% of all malignant tumours[6]. In the last decades of the 20th century, 5-year relative survival for renal cancer patients was about 55% in Europe[7] and 60% in the United States[8]. The disease is more frequent in men and in rural areas[6].

The incorporation of new diagnostic techniques in recent years, such as echography, computed tomography, and magnetic resonance imaging, may have had an influence on the detected rise in incidence[1]. Nevertheless, the increased use of these imaging procedures does not completely account for the rising incidence of all stages of kidney cancer[9], and other well-known risk factors, such as smoking, may also be contributing to the underlying incidence and mortality trends. To explore recent trends in Europe, this study analysed and compared trends in kidney cancer mortality in different European countries over the last two decades, quantifying the effect of age, death period, and birth cohort on the observed trends.

## Methods

The number of kidney cancer deaths and the population estimates for the respective European countries, broken down by year, sex, and age group, were drawn from the World Health Organization Mortality Database over the period 1981–2000. Deaths from kidney cancer corresponded to code 189 of the eighth and ninth revisions of the International Classification of Diseases[10], and codes C64- C66, C68 of the tenth revision[11]. Countries of the former Czechoslovakia, USSR, and Yugoslavia were excluded from the analysis since data were only available as a whole during the 1980 decade. Similarly, Germany was excluded to avoid spurious trends resulting from merging data of the former Democratic and Federal Republics prior to 1990. Some countries, such as Belgium, Poland, Portugal, Romania, and Switzerland, were also excluded due to incomplete kidney cancer mortality series during the study period. For the remaining countries, matrices of kidney cancer deaths and population broken down by 5-year calendar period and age group (0–4, 5–9,..., 80–84, and ≥ 85 years) were generated.

Overall age- and period-adjusted mortality rates, as well as 5-year period (1981–1985 to 1996–2000) age-adjusted rates, were calculated for each country and sex using the direct method with the European standard population. Log-linear Poisson models were fitted to obtain the annual percentage change (APC) in age-adjusted mortality rates for each country. Under the assumption that the number of deaths by country, 5-year period, and age group had a Poisson distribution, estimates and 95% confidence intervals (CIs) of age- and period-adjusted mortality ratios were computed for each country taking the average for all European countries as reference.

To further study the existence of regional patterns in kidney cancer mortality, countries were grouped in four geographical regions: North (Denmark, Finland, Norway, and Sweden), West (Austria, France, Ireland, Netherlands, and United Kingdom), East (Bulgaria and Hungary), and South (Greece, Italy, and Spain). With data aggregated by region, the same methods described above were used to estimate the APC in age-adjusted mortality rates, as well as the age- and period-adjusted mortality ratio for each region compared to the southern European countries, this latter region being taken as reference on registering the lowest rates.

Finally, for each country, log-linear Poisson models were fitted to study the effect of age, period of death, and birth cohort. Given the 'non-identifiability' problem (the three factors age, period, and cohort are linearly dependent), we used Osmond-Gardner's[12] and Decarli-La Vecchia's[13] solutions, as well as the evaluation of estimable parameters proposed by Holford[14], such as the degree of curvature in each effect and the sum of period and cohort linear slopes, also known as the net drift. The Osmond-Gardner and Decarli-La Vecchia solutions split the net drift into cohort and period slopes by minimizing the disagreement in parameter estimates between the full three-factor model and each of the two-factor models (age period, age cohort, and period cohort). To graphically display the individual effects, we applied both solutions, together with the extreme alternative solutions determined by curvatures and exclusive cohort or period linear effects (that is, assuming zero period or cohort slope). Age groups younger than 40 years in men, as well as those younger than 45 years in women, were excluded from this analysis due to the limited number of deaths in these age groups for most countries. The open-ended category of persons aged 85 years and older was also excluded, so that age-period-cohort analyses were restricted to ages 40–84 years in men and 45–84 years in women. We checked for extra-Poisson dispersion[15], and where this was present, effects were calculated using a negative binomial distribution. Significant curvatures in cohort and period effects were tested by using the null chi-square distribution of the corresponding likelihood ratio statistics. Specific functions for these analyses were written in R[16].

## Results

### Mortality rates by country

Table 1 shows age- and period-adjusted male and female mortality rates for the different countries over the entire 1981–2000 period. In all countries studied, mortality was higher in men than in women. The male-to-female rate ratio was smaller in most Nordic countries (1.59 in Denmark and 1.72 in Sweden), and greater in the southern countries (2.63, 2.61, and 2.47 in Italy, Spain, and Greece, respectively).

Among men, the highest age- and period-adjusted mortality rates were registered by Hungary, Austria, and the Nordic countries (rate ratio with respect to the average for European countries of 1.48 in Hungary, 1.39 in Austria, 1.34 in Finland, and 1.32 in Sweden), and the lowest rates by Bulgaria, Greece, and Spain (rate ratios of 0.51, 0.62, and 0.69, respectively) (Table 1). However, when country-specific trends were examined (Table 2), it was the Nordic countries that registered the most favourable results. The trend was significantly downward in Denmark, Sweden, and Austria (APC -1.0%, -0.9%, and -0.5%, respectively) and borderline but non-significant in Finland and Norway. All the remaining countries displayed significant rising trends. The highest annual increases were mainly observed in the southern and eastern European countries (APC 3.0% in Spain, 2.5% in Bulgaria, 2.1% in Greece, and 1.9% in Hungary).

For women, the results were similar to those observed among men. The highest age- and period-adjusted mortality rates corresponded to the Nordic countries and Austria (rate ratio with respect to the average for European countries of 1.82 in Denmark, 1.74 in Sweden, 1.60 in Austria, and 1.54 in Finland), while the lowest rates were registered by Bulgaria, Greece, and Spain (rate ratios of 0.54, 0.57, and 0.60, respectively) (Table 1). The Nordic countries displayed downward trends, that were statistically significant for Denmark and Sweden (APC -1.4% and -1.1%, respectively), whereas the most steeply rising trends were observed in the southern and eastern European countries (APC 2.4% in Greece, 2.1% in Spain, 1.7% in Hungary, and 1.5% in Bulgaria) (Table 2).

### Mortality rates by geographical region

Analyses by region confirmed a clear north-south pattern. Compared to men in the southern region, overall age- and period-adjusted mortality rates were 20% higher in the eastern region (95% CI 17 to 22%), 25% higher in the west (95% CI 24 to 27%), and 53% higher in the north (95% CI 50 to 55%) (Table 3). Regarding trends, age-adjusted mortality rates showed a significant annual decrease of -0.7% among men in the north of Europe (95% CI -0.9 to -0.4%), a moderate rise of 0.7% in the west (95% CI 0.6 to 0.8%), and substantial increases of 1.4% in the south (95% CI 1.2 to 1.5%) and 2.0% in the east (95% CI 1.6 to 2.3%) (Table 4). The pattern was similar among women (Tables 3 and 4), yet some greater differences were observed across regions, with a north-to-south rate ratio of 2.21 (95% CI 2.17 to 2.26). Annual increases in south and east regions were slightly lower (0.9% and 1.6%, respectively) than those registered for men.

### Age-period-cohort effect

**Figure 1:**
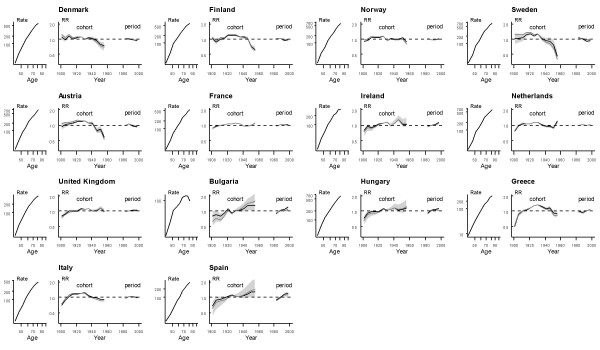
Age, period, and cohort effects on kidney cancer mortality for European men. Age effects are expressed as rates per 1,000,000 person-years, and period and cohort effects as rate ratios (RR) with respect to their average. Osmond-Gardner (black lines) and Decarli-La Vecchia solutions (grey lines) were used to estimate period and cohort effects. The shaded areas are determined by curvatures and exclusive cohort or period slopes. See Methods for further details.

**Figure 2:**
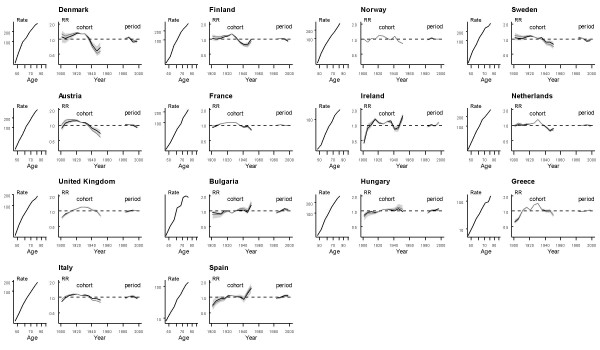
Age, period, and cohort effects on kidney cancer mortality for European women. Age effects are expressed as rates per 1,000,000 person-years, and period and cohort effects as rate ratios (RR) with respect to their average. Osmond-Gardner (black lines) and Decarli-La Vecchia solutions (grey lines) were used to estimate period and cohort effects. The shaded areas are determined by curvatures and exclusive cohort or period slopes. See Methods for further details.

When age-period-cohort models were fitted to each country, similar results were obtained under both Osmond-Gardner and Decarli-La Vecchia solutions, and in those countries with low net drifts, they also agreed with the exclusive effects. Among men (Figure 1), three patterns of evolution were observed for cohort effects: a first group with the Nordic countries, in which mortality trend was flat or slightly upward to cohorts born around 1920 and downward thereafter (*p *< 0.001 for curvature in cohort effect); a second group comprised mainly of western and southern European countries, in which mortality rose until cohorts born around 1930, and then began to decline thereafter (*p *< 0.001 for cohort curvature); and a third group with the eastern European countries, in which mortality increased gradually throughout all birth cohorts, with no clear evidence of a downward trend for the youngest generations (*p *= 0.043 for cohort curvature). Similar but more heterogeneous regional patterns were observed for period effects, with a downward trend in the Nordic countries (*p *= 0.348 for period curvature) and an upward trend in the eastern countries (*p *= 0.104 for period curvature). Among women (Figure 2), the shape of cohort and period effects was in general very similar to that observed for men. Significant departures from the linear trend in cohort effects were detected among women in the Nordic, western, and southern countries (*p *< 0.001 for cohort curvatures), but not in the eastern countries (*p *= 0.914 for cohort curvature).

## Discussion

Our results show a significant north-south pattern in kidney cancer mortality during the last two decades, with higher rates on a downward trend in the northern European countries and lower rates on an upward trend in the southern countries. Age-period-cohort models also suggest that mortality rates decreased for those cohorts born after 1920 in the Nordic countries, while the trend began to decline for younger generations born around 1930 in the western and southern European countries. For eastern countries, however, rates appeared to increase continuously throughout all birth cohorts.

Renal cell cancer has two forms of presentation, one hereditary and the other sporadic[17]. The aetiology is not completely understood. Besides smoking, which is the most widely accepted risk factor [18-21], there are other factors that might be associated with increased risk of kidney cancer. Environmental exposure to uranium has shown no direct influence on kidney cancer, even though uranium deposited in the human body was related to renal toxicity[22]. The role of regular use of analgesics and diuretics are controversial aspects[3,6,23]. Different studies have reported an association between development of kidney cancer and several occupational exposures, such as work in laundries and printing plants, as well as exposure to chemical substances, principally liposoluble solvents, with this association being stronger in women [24-26]. Other risk factors include obesity and hypertension[18,27-29], as well as certain renal disorders and chronic haemodialysis[3,6]. A protective effect has been observed with frequent intake of vegetables in both men and women[30] and with moderate alcohol consumption in women[29,31], but no consistent association has been found with physical activity[32,33].

In this study, kidney cancer mortality trends among European men are substantially similar to those already described for lung cancer[34,35], in great part attributable to smoking, the principal known risk factor for both kidney and lung cancers. The different birth-cohort patterns observed in kidney cancer mortality among European countries may largely be attributed to the different smoking patterns. In the Nordic countries, the upward trend becomes inverted with the decline in smoking prevalence, that was mainly due to higher proportions of never smokers in the younger generations[36]. The levelling off of kidney cancer mortality rates takes place in a lagged fashion for those countries in the west and south of Europe that undergo the shift in tobacco consumption at a later point in time. In the eastern European countries, however, the mortality trend is upward as a consequence of the increasing prevalence of smokers in successive birth cohorts[36].

In addition to the effect of smoking, discussion about this tumour's evolution tends to focus on whether diagnostic and therapeutic improvements might contribute to explain the underlying mortality trend. It has been shown that the increased use of new imaging diagnostic procedures brings forward the date of diagnosis in many renal cancer patients, thus increasing the incidence of localized and presymptomatic tumours that otherwise would have been diagnosed at more advanced disease stages[1]. This early detection, together with the modest benefit of immunotherapy for advanced renal cell cancer[37], have contributed to improve the overall European 5-year survival for kidney cancer by about 12% from the 1980 to 1990 decades[7,38]. This improvement in prognosis is consistent with the initial upward and subsequent downward period effect on kidney cancer mortality displayed for both sexes in some European countries. Nevertheless, diagnostic and therapeutic improvements cannot fully explain the heterogeneity in period effects across Europe. Other alternative factors, such as the different impact of public health interventions on smoking cessation, may also be contributing to the observed regional variation.

## Conclusion

In summary, substantial differences in kidney cancer mortality were observed across Europe over the last two decades of the 20th century, with high rates on a downward trend in the north, intermediate rates on a more marked rising trend in the east than in the west, and low rates on an upward trend in the south. The downward pattern observed for those cohorts born after 1920–1930 in northern, western, and southern regions suggests more favourable trends in coming years, in contrast to the eastern countries where birth-cohort pattern remains upward.

## Competing interests

The author(s) declare that they have no competing interests.

## Authors' contributions

NPF, GLA, and RPB have made substantial contributions to study conception, statistical analysis, and interpretation of results, as well as in drafting and revising the manuscript. The three authors read and approved the final manuscript.

## Pre-publication history

The pre-publication history for this paper can be accessed here:



## Figures and Tables

**Table 1:** Age- and period-adjusted kidney cancer mortality rates (per European standard 100,000 population) over the entire 1981–2000 period by country and sex.

	Men				Women				
			
Country	Rate	RR*	95% CI	*p*	Rate	RR*	95% CI	*p*	RR†
North									
Denmark	7.62	1.27	1.23 to 1.31	<0.001	4.80	1.82	1.76 to 1.88	<0.001	1.59
Finland	8.04	1.34	1.30 to 1.39	<0.001	4.07	1.54	1.49 to 1.60	<0.001	1.97
Norway	7.16	1.19	1.15 to 1.24	<0.001	3.47	1.32	1.26 to 1.38	<0.001	2.06
Sweden	7.92	1.32	1.29 to 1.35	<0.001	4.59	1.74	1.69 to 1.79	<0.001	1.72
West									
Austria	8.32	1.39	1.35 to 1.42	<0.001	4.23	1.60	1.56 to 1.65	<0.001	1.97
France	6.60	1.10	1.09 to 1.11	<0.001	2.64	1.00	0.99 to 1.01	0.957	2.50
Ireland	4.99	0.83	0.79 to 0.88	<0.001	2.38	0.90	0.84 to 0.97	0.004	2.09
Netherlands	7.87	1.31	1.29 to 1.34	<0.001	3.91	1.48	1.45 to 1.52	<0.001	2.01
UK	5.63	0.94	0.93 to 0.95	<0.001	2.63	1.00	0.98 to 1.01	0.522	2.14
East									
Bulgaria	3.04	0.51	0.49 to 0.53	<0.001	1.43	0.54	0.52 to 0.57	<0.001	2.12
Hungary	8.90	1.48	1.45 to 1.52	<0.001	3.95	1.50	1.46 to 1.54	<0.001	2.25
South									
Greece	3.73	0.62	0.60 to 0.64	<0.001	1.51	0.57	0.55 to 0.60	<0.001	2.47
Italy	5.92	0.99	0.98 to 1.00	0.010	2.26	0.85	0.84 to 0.87	<0.001	2.63
Spain	4.14	0.69	0.68 to 0.70	<0.001	1.59	0.60	0.59 to 0.61	<0.001	2.61

* Rate ratio with respect to the average for all European countries.

† Male to female rate ratio.

CI, confidence interval; UK, United Kingdom.

**Table 2:** Age-adjusted kidney cancer mortality rates (per European standard 100,000 population) in 5-year periods by country and sex.

	Mortality rate						
				
Country	1981–1985	1986–1990	1991–1995	1996–2000	APC (%)*	95% CI	*p*
Men							
North							
Denmark	8.20	7.87	7.37	7.02	-1.0	-1.6 to -0.5	<0.001
Finland	8.10	8.32	7.84	7.91	-0.3	-0.9 to 0.3	0.358
Norway	7.40	7.00	6.97	7.27	0.0	-0.6 to 0.6	0.929
Sweden	8.65	8.24	7.47	7.31	-0.9	-1.3 to -0.5	<0.001
West							
Austria	8.20	8.93	8.44	7.73	-0.5	-0.9 to 0.0	0.036
France	6.11	6.80	6.71	6.79	0.6	0.4 to 0.8	<0.001
Ireland	4.23	4.66	5.21	5.86	2.0	1.1 to 3.0	<0.001
Netherlands	7.42	8.05	7.93	8.09	0.4	0.1 to 0.8	0.013
UK	5.16	5.57	5.87	5.93	0.9	0.7 to 1.1	<0.001
East							
Bulgaria	2.44	2.93	3.17	3.60	2.5	1.8 to 3.2	<0.001
Hungary	7.30	8.80	9.57	9.94	1.9	1.5 to 2.3	<0.001
South							
Greece	3.33	3.39	3.90	4.32	2.1	1.5 to 2.6	<0.001
Italy	5.31	6.18	6.33	5.89	0.6	0.4 to 0.8	<0.001
Spain	3.15	3.84	4.60	4.96	3.0	2.7 to 3.3	<0.001
Women							
North							
Denmark	5.26	5.42	4.39	4.15	-1.4	-1.9 to -0.8	<0.001
Finlandia	4.17	4.26	4.20	3.67	-0.5	-1.1 to 0.2	0.146
Norway	3.40	3.62	3.35	3.52	0.4	-0.3 to 1.2	0.286
Sweden	5.05	4.91	4.18	4.23	-1.1	-1.6 to -0.7	<0.001
West							
Austria	4.48	4.32	4.25	3.86	-0.3	-0.8 to 0.2	0.176
France	2.58	2.68	2.66	2.64	0.5	0.2 to 0.7	<0.001
Ireland	2.20	2.38	2.36	2.60	1.4	0.2 to 2.6	0.023
Netherlands	3.95	3.98	3.96	3.77	-0.2	-0.6 to 0.3	0.469
UK	2.36	2.62	2.84	2.70	1.4	1.2 to 1.6	<0.001
East							
Bulgaria	1.26	1.35	1.56	1.56	1.5	0.6 to 2.4	0.001
Hungary	3.42	3.88	4.08	4.43	1.7	1.2 to 2.2	<0.001
South							
Greece	1.31	1.41	1.57	1.74	2.4	1.6 to 3.2	<0.001
Italy	2.16	2.35	2.38	2.13	0.2	0.0 to 0.5	0.081
Spain	1.35	1.49	1.71	1.79	2.1	1.7 to 2.5	<0.001

* Average annual percentage change in age-adjusted mortality rates.

CI, confidence interval; UK, United Kingdom.

**Table 3:** Age- and period-adjusted kidney cancer mortality rates (per European standard 100,000 population) over the entire 1981–2000 period by geographical region and sex.

	Men				Women				
			
Region	Rate	RR*	95% CI	*p*	Rate	RR*	95% CI	*p*	RR†
North	7.76	1.53	1.50 to 1.55	<0.001	4.33	2.21	2.17 to 2.26	<0.001	1.79
West	6.37	1.25	1.24 to 1.27	<0.001	2.85	1.46	1.44 to 1.48	<0.001	2.24
East	6.08	1.20	1.17 to 1.22	<0.001	2.87	1.47	1.43 to 1.51	<0.001	2.12
South	5.08	1	-	-	1.95	1	-	-	2.60

* Rate ratio with respect to the southern European region.

† Male to female rate ratio.

CI, confidence interval.

**Table 4:** Age-adjusted kidney cancer mortality rates (per European standard 100,000 population) in 5-year periods by geographical region and sex.

	Mortality rate						
				
Region	1981–1985	1986–1990	1991–1995	1996–2000	APC (%)*	95% CI	*P*
Men							
North	8.23	7.97	7.46	7.37	-0.7	-0.9 to -0.4	<0.001
West	5.90	6.47	6.53	6.58	0.7	0.6 to 0.8	<0.001
East	5.00	5.98	6.45	6.90	2.0	1.6 to 2.3	<0.001
South	4.37	5.09	5.48	5.40	1.4	1.2 to 1.5	<0.001
Women							
North	4.62	4.65	4.08	3.96	-0.8	-1.1 to -0.5	<0.001
West	2.72	2.87	2.95	2.84	0.7	0.5 to 0.8	<0.001
East	2.51	2.79	2.99	3.20	1.6	1.1 to 2.0	<0.001
South	1.80	1.97	2.07	1.97	0.9	0.7 to 1.1	<0.001

* Average annual percentage change in age-adjusted mortality rates.

CI, confidence interval.

## References

[B1] Chow WH, Devesa SS, Warren JL, Fraumeni JF (1999). Rising incidence of renal cell cancer in the United States. JAMA.

[B2] Lopez-Abente G, Pollan M, Vergara A, Ardanaz E, Moreo P, Moreno C, Ruiz M (2000). Tendencia temporal de la incidencia de cáncer en Navarra y Zaragoza. Gac Sanit.

[B3] Whang YE, Godley PA (2003). Renal cell carcinoma. Curr Opin Oncol.

[B4] Mathew A, Devesa SS, Fraumeni JF, Chow WH (2002). Global increases in kidney cancer incidence, 1973–1992. Eur J Cancer Prev.

[B5] Ferlay J, Bray F, Sankila R, Parkin DM (1999). EUCAN: Cancer incidence, mortality and prevalence in the European Union version 50 IARC CancerBase No 4.

[B6] Marston W, Shipley WU, Parkinson DR, De Vita VT, Hellman S, Rosenberg SA (1997). Cancer of the kidney and ureter. Cancer principles & practice of oncology.

[B7] Sant M, Aareleid T, Berrino F, Bielska LM, Carli PM, Faivre J, Grosclaude P, Hedelin G, Matsuda T, Moller H, Moller T, Verdecchia A, Capocaccia R, Gatta G, Micheli A, Santaquilani M, Roazzi P, Lisi D (2003). EUROCARE-3: survival of cancer patients diagnosed 1990–94-results and commentary. Ann Oncol.

[B8] Brenner H (2002). Long-term survival rates of cancer patients achieved by the end of the 20th century: a period analysis. Lancet.

[B9] Hock LM, Lynch J, Balaji KC (2002). Increasing incidence of all stages of kidney cancer in the last 2 decades in the United States: an analysis of surveillance, epidemiology and end results program data. J Urol.

[B10] World Health Organization (1977). International Classification of Diseases, 9th Revision.

[B11] World Health Organization (1992). International Statistical Classification of Diseases and Related Health Problems, 10th Revision.

[B12] Osmond C, Gardner MJ (1982). Age, period and cohort models applied to cancer mortality rates. Stat Med.

[B13] Decarli A, La Vecchia C (1987). Age, period and cohort models: review of knowledge and implementation in GLIM. Riv Stat Applicata.

[B14] Holford TR (1991). Understanding the effects of age, period, and cohort on incidence and mortality rates. Annu Rev Public Health.

[B15] Dean CB (1992). Testing for overdispersion in Poisson and binomial regression models. J Am Stat Assoc.

[B16] Ihaka R, Gentleman RR (1996). R: A language for data analysis and graphics. J Comput Appl Math.

[B17] Gago-Dominguez M, Yuan JM, Castelao JE, Ross RK, Yu MC (2001). Family history and risk of renal cell carcinoma. Cancer Epidemiol Biomarkers Prev.

[B18] Tavani A, La Vecchia C (1997). Epidemiology of renal-cell carcinoma. J Nephrol.

[B19] La Vecchia C, Negri E, Levi F, Decarli A, Boyle P (1998). Cancer mortality in Europe: effects of age, cohort of birth and period of death. Eur J Cancer.

[B20] Parker AS, Cerhan JR, Janney CA, Lynch CF, Cantor KP (2003). Smoking cessation and renal cell carcinoma. Ann Epidemiol.

[B21] Levi F, Lucchini F, Negri E, La Vecchia C (2004). Declining mortality from kidney cancer in Europe. Ann Oncol.

[B22] Taylor DM, Taylor SK (1997). Environmental uranium and human health. Rev Environ Health.

[B23] Gago-Dominguez M, Yuan JM, Castelao JE, Ross RK, Yu MC (1999). Regular use of analgesics is a risk factor for renal cell carcinoma. Br J Cancer.

[B24] Parent ME, Hua Y, Siemiatycki J (2000). Occupational risk factors for renal cell carcinoma in Montreal. Am J Ind Med.

[B25] Pesch B, Haerting J, Ranft U, Klimpel A, Oelschlagel B, Schill W (2000). Occupational risk factors for renal cell carcinoma: agent-specific results from a case-control study in Germany. MURC Study Group. Multicenter urothelial and renal cancer study. Int J Epidemiol.

[B26] Dosemeci M, Cocco P, Chow WH (1999). Gender differences in risk of renal cell carcinoma and occupational exposures to chlorinated aliphatic hydrocarbons. Am J Ind Med.

[B27] Chow WH, Gridley G, Fraumeni JF, Jarvholm B (2000). Obesity, hypertension, and the risk of kidney cancer in men. N Engl J Med.

[B28] Calle EE, Rodriguez C, Walker-Thurmond K, Thun MJ (2003). Overweight, obesity, and mortality from cancer in a prospectively studied cohort of U.S. adults. N Engl J Med.

[B29] Nicodemus KK, Sweeney C, Folsom AR (2004). Evaluation of dietary, medical and lifestyle risk factors for incident kidney cancer in postmenopausal women. Int J Cancer.

[B30] Hu J, Mao Y, White K (2003). Diet and vitamin or mineral supplements and risk of renal cell carcinoma in Canada. Cancer Causes Control.

[B31] Parker AS, Cerhan JR, Lynch CF, Ershow AG, Cantor KP (2002). Gender, alcohol consumption, and renal cell carcinoma. Am J Epidemiol.

[B32] Bergstrom A, Terry P, Lindblad P, Lichtenstein P, Ahlbom A, Feychting M, Wolk A (2001). Physical activity and risk of renal cell cancer. Int J Cancer.

[B33] Mahabir S, Leitzmann MF, Pietinen P, Albanes D, Virtamo J, Taylor PR (2004). Physical activity and renal cell cancer risk in a cohort of male smokers. Int J Cancer.

[B34] Levi F, Randimbison L, La Vecchia C, Erler G, Te VC (1998). Multiple primary cancers to indicate associations between smoking and cancer incidence: Vaud and Neuchatel, Switzerland, 1974–1994. Int J Cancer.

[B35] Lopez-Abente G, Pollan M, de la Iglesia P, Ruiz M (1995). Characterization of the lung cancer epidemic in the European Union (1970–1990). Cancer Epidemiol Biomarkers Prev.

[B36] Molarius A, Parsons RW, Dobson AJ, Evans A, Fortmann SP, Jamrozik K, Kuulasmaa K, Moltchanov V, Sans S, Tuomilehto J, Puska P (2001). Trends in cigarette smoking in 36 populations from the early 1980s to the mid-1990s: findings from the WHO MONICA Project. Am J Public Health.

[B37] Coppin C, Porzsolt F, Awa A, Kumpf J, Coldman A, Wilt T (2005). Immunotherapy for advanced renal cell cancer. Cochrane Database Syst Rev.

[B38] Berrino F, Capocaccia R, Estève J, Gatta G, Hakulinen T, Micheli A, Sant M, Verdecchia A (1999). Survival of cancer patients in Europe: the EUROCARE-2 Study.

